# The Bare Past

**DOI:** 10.1007/s11406-022-00534-8

**Published:** 2022-06-08

**Authors:** Vincent Grandjean

**Affiliations:** Faculty of Philosophy, Radcliffe Humanities, University of Oxford, Radcliffe Observatory Quarter, Woodstock Road, OX2 6GG Oxford, UK

**Keywords:** Growing Block Theory, Bare particulars, Natural kind, Existence in the past, Epistemic objection, Essentialism

## Abstract

In this paper, I first introduce one of the most prominent objections against the Growing Block Theory of time (GBT), the so-called ‘epistemic objection’, according to which GBT provides no way of knowing that our time is the objective present and, therefore, leads at best to absolute skepticism about our temporal location, at worst to the quasi-certainty that we are located in the objective past. Secondly, I express my dissatisfaction regarding the various traditional attempts to address this objection, especially Merricks (2006), Forrest (2004) and Correia and Rosenkranz (2018). Thirdly, I show that the passage of time leads to an anti-essentialist picture of natural kinds. Finally, I develop my own solution to the epistemic objection, based on the continued existence of bare particulars.

## Introduction

One can distinguishes at least two ways of doing philosophy of time. First, one can be interested in the phenomenology of temporal awareness. Edmund Husserl (1964), for example, argues that a perspicuous account of time must explain *how* temporal objects (e.g., a melody) though composed of distinguishable moments, are apprehended as a unity (rather than as a convoluted patchwork). Secondly, one can be interested in physical time, which belongs to the reality itself. For example, Vesselin Petkov (2006) argues for the ‘block universe’ view of time, according to which time is an ingredient of a single, four-dimensional relativistic spacetime. The Growing Block Theory (‘GBT’ thereafter) falls more to this second way of doing philosophy of time, since it describes time as much an objective dimension of reality as any of the three spatial ones. However, most proponents of GBT break away from this traditional distinction, as they try to reconcile the phenomenology of temporal awareness with physical time. In short, they often take some of our pre-theoretical intuitions (e.g., the intuition that time passes, that the future is open, etc.) to be reliable indicators of *how* physical time actually is (cf. Grandjean 2021a).

GBT was first set out in C.D. Broad’s *Scientific Thought* (1923). This theory is commonly introduced as a hybrid between presentism (i.e. the theory that only the present exists) and eternalism (i.e. the theory that the past, the present, and the future exist). For, GBT is committed to the existence of the past (and the present), but not to the existence of the future. Moreover, GBT is a *dynamic* theory: it depicts the block universe as increasing, with new moments of times coming into existence to join the moments that already exist. As Broad puts it: “[n]othing has happened to the present by becoming past except that fresh slices of existence have been added to the total history of the world. The past is thus as real as the present. On the other hand, the essence of a present event is, not that it precedes future events, but that there is quite literally *nothing* to which it has the relation of precedence” (1923: 66). Since, from Broad’s perspective, there is nothing such as ceasing to exist, the sum total of existence is always increasing. Finally, GBT takes the present to be the ‘boundary of reality’, beyond which there is nothing at all. To use William Grey’s metaphor, the present is a kind of “ontological gateway” through which times and events have to pass on their way to become real and always remain so (cf. 1997: 216).

However, despite some attractive features,^1^ GBT is subject to many objections. In particular, since GBT is commonly introduced as a hybrid between presentism and eternalism, it is often criticized for accumulating the flaws that are identified in these two mainstream theories. For example, just like presentism, GBT seems to conflict with the theories of relativity, especially because of the absolute notion of objective simultaneity it requires to define the ‘boundary of reality’ (cf. Rietdijk, 1966, Putnam, 1967). Furthermore, just like the ‘moving spotlight’ view, which corresponds to the A-theoretic version of eternalism, GBT seems to face with the so-called ‘epistemic objection’, according to which it would provide no way of knowing that our time is the objective present.^2^ The present article deals only with the latter objection. In the second section, I will expose the epistemic objection, as well as some immediate attempts to escape it. In the third section, I will introduce three more substantive ways of responding the objection; I will discuss the solutions to be found in Trenton Merricks (2006), Peter Forrest (2004), and Fabrice Correia and Sven Rosenkranz (2018). In the fourth section, I will show that the passage of time leads to an anti-essentialist picture of natural kinds. Finally, in the fifth section, I will develop my own solution to the epistemic objection, based on the continued existence of bare particulars.

## A Skeptical Challenge for GBT

One of the most prominent objections against GBT, commonly called ‘the epistemic objection’, was first pressed by Craig Bourne (2002) and further developed by David Braddon-Mitchell (2004) and Trenton Merricks (2006). In its original form, this objection purports to show that GBT leads to absolute skepticism about *where* we are temporally located. In particular, GBT would provide no basis for saying that our time is the objective present. But it is also possible to sharpen the epistemic objection in order to obtain an even more problematic conclusion: GBT would imply that we are, almost certainly, located in the objective past. In other words, the growing block’s edge is most likely located in the future of now. The epistemic objection can be formulated as follows.

According to GBT, both the past and the present exist and, therefore, everything that is either past or present exists. For example, Napoleon exists (although he is not located at the present time), as well as everything that concerns Napoleon, such as his beliefs. Among Napoleon’s beliefs is presumably the belief that he is located in the present when, for example, he is crowning the Emperor.^3^ It indeed seems that, just as we believe to be located in the present at the current moment, Napoleon believes that he is located in the present when he becomes the first Emperor of the French. In other words, GBT seems to imply not only that thinking subjects located in the objective past exist, but also that they believe that the time they exist at is the objective present. Yet, we obviously know that these thinking subjects are wrong in holding this belief, since we succeed them. In particular, we have no doubt that Napoleon is located in the past (he died two hundred years ago, after all). Of course, that does not mean that Napoleon has never been right to believe that he was located in the present: his belief was true on 2 December 1804, but is clearly false on 20 April 2022.

Taking that for granted, the question that Craig Bourne (2002), David Braddon-Mitchell (2004) and Trenton Merricks (2006) have asked to growing blockers is: ‘Assuming that GBT is true, what guarantees that *we*, as thinking subjects, are located in the present?’. Perhaps, in the year 2120, some people look at us just as we look at Napoleon, and think: ‘These naive people believe that they are located in the present, while they are embedded in the past!’. In other words, we are in no better epistemic position than thinking subjects located in the objective past who are wrongly believing that they are located in the objective present, since “[…] we would have all the same beliefs […] even if we were past” (Bourne, 2002: 362). GBT seems therefore to lead to absolute skepticism about our temporal localization. Specifically, this theory seems to provide no reason to believe that we are located in the present time.

Worse, the probability that our time is the objective present (and therefore that we are right to believe that it is) is vanishingly small. After all, the objective present might well be located tomorrow, next year, or five billion years beyond the current moment, so that we are actually located in the objective past. Since all these alternatives should be regarded as equally likely (our beliefs would in each case be the same, after all), the hypothesis that our time is the objective present is almost certainly false (cf. Braddon-Mitchell, 2004: 200). In other words, the only possibility that we are located in the present does not carry any weight (in terms of probability) in the face of the multitude of possibilities that we are actually located in the past, so that we can assert without a doubt that we are living in the past. These two implications – skepticism about our temporal localization, and the quasi-certainty of being localized in the past – look absurd and must, according to some, lead us to reject GBT.

However, there are some immediate reasons to think that this objection is not *definitive*. First, it may be noted that there is something paradoxical in arguing that GBT leads to absolute skepticism about *where* we are temporally located and, at the same time, that GBT implies that we are (almost certainly) located in the objective past. It seems that these two implications cannot both be true: either a theory is guilty of generating doubts, or it is guilty of generating counterintuitive certitudes. But, clearly, if GBT implies that we are (almost certainly) located in the objective past, then it is simply wrong to claim that this theory provides no basis for knowing *where* we are temporally located: we are in the past, there is (almost) no doubt about this! Of course, one could reply that, although GBT implies that we are (almost certainly) located in the objective past, this does not rule out any form of skepticism; proponents of the epistemic objection seem mainly concerned with our *exact* location. For example, it might be argued that GBT provides no basis for saying whether we are located in the recent or the distant past. But, even if one concedes this, it is still wrong to claim that we have no way of knowing that our time is (or, in this case, is not) the objective present. Obviously, this preliminary remark does not put an end to the debate since, even taken on an individual basis, these two implications remain problematic for GBT. But it indicates that the epistemic objection might rest on some sophistic premises that may (and will) be challenged.

Secondly, it may be noted that the epistemic objection does not merely concern GBT, but is equally applicable to every A-theory of time that distinguishes between the notions of *existing at the present time* and just *existing*. For example, the epistemic objection is equally applicable to the moving spotlight theorists (who combine eternalism and the A-theory of time). In a certain sense, the problem is even more serious for them, since not only could their theory imply an infinite number of possibilities that we are located in the objective past, but also an infinite number of possibilities that we are located in the objective future. In an A-eternalist context, the probability of being localized in the objective present is therefore even lower than in GBT. By contrast, presentism seems to be immune to the epistemic objection: if no other times exist, then there is no puzzle in knowing that we are currently in the objective present (cf. Bourne, 2006: 24, Braddon-Mitchell, 2004: 199, Heathwood, 2005: 50, Zimmerman, 2008: 216).^4^ Of course, pointing out the flaws of other theories does not put GBT in a better position; but it shows at least that if we take the epistemic objection seriously, then we are compelled either to accept presentism or to abandon the idea of an objective present, which sounds suspicious. It would indeed be quite surprising that such a skeptical challenge, though embarrassing, could undermine any attempt to defend a non-presentist A-theory of time, even though sometimes some facts are indeed surprising.

Thirdly, one could regard the epistemic objection as completely harmless. After all, as its name suggests, this objection is of an epistemic nature and, therefore, cannot pose a threat to GBT, which is a metaphysical theory. In that sense, GBT may perhaps lead to absolute skepticism about *where* we are temporally located (or even to the quasi-certainty that we all are temporally located in the past), but this does not imply that GBT is false. What we can (or cannot) know about our temporal location has no impact on the temporal structure of the world. Assuming that the world is such as growing blockers claim, we might have no choice but to accept skepticism as an unfortunate consequence. This reply sounds acceptable, but since one of the main reasons for accepting GBT is that it can account for some pre-theoretic intuitions we have regarding the nature of time, it would surely be problematic to end up with a theory that implies the truth of positions as counterintuitive as skepticism. Worse than this: if the skeptical conclusion is accepted, the openness intuition for accepting GBT in the first place is undermined, because the future is open only when one is at the present – if I am deep into the past of the block, the future (or at least my immediate future for as long as the present is in my future) is closed. It therefore seems preferable to favor more substantial solutions to the skeptical challenge raised by the epistemic objection, such as those developed by Trenton Merricks (2006), Peter Forrest (2004), or Fabrice Correia and Sven Rosenkranz (2018).

## Three Unsatisfactory Attempts to Meet the Skeptical Challenge

A first solution to the skeptical challenge is due to Trenton Merricks (2006), who argues that the epistemic objection relies on an uncharitable interpretation of what beliefs, such as ˹I am sitting here at the present time˺, are about. After all, assuming that a belief about the present cannot occur instantaneously, the growing blocker might have no choice but to concede that such beliefs are never (not even for an instant) true, which sounds implausible. Merricks therefore proposes to distinguish the ‘*objective* present’ (i.e. the growing edge of reality) from the ‘*subjective* present’ (i.e. an indexical, like ‘here’ or ‘this place’). According to him, growing blockers should say that Napoleon’s beliefs like ˹I am crowning the Emperor at the present time˺ are always about the *subjective* present, so that such beliefs can be true even though Napoleon is not on the edge of reality. Similarly, growing blockers should say that everyone else’s beliefs about ‘the present’ are in fact beliefs about the *subjective* present, which prevents GBT from implying that every belief about the present is almost certainly false. In brief, Merricks argues that GBT should be regarded as a hybrid between the A- and the B-theory of time: growing blockers should agree with B-theorists that ‘the present’ is typically an indexical (i.e. the present is typically *a matter of perspective*), and they should agree with A-theorists that, in addition, there is an *objective* present (the growing edge of reality) used when GBT itself is being discussed.

However, as Merricks himself points out, the distinction between the ‘*objective* present’ and the ‘*subjective* present’ detracts GBT from its original purpose: to provide a natural view of time. The distinction between the ‘*objective* present’ and the ‘*subjective* present’ calls for other distinctions, especially between the ‘*objective* future’ (i.e. the time which is not yet part of being) and the ‘*subjective* future’ (i.e. the future that follows the *subjective* present).^5^ In particular, growing blockers must acknowledge that, just as our typical beliefs about the present are in fact beliefs about the *subjective* present, our typical beliefs about the future are in fact beliefs about the *subjective* future. For example, we are certainly right to believe that ˹the discovery of a cure for cancer is in the future˺, provided that by ‘the future’ we mean ‘the *subjective* future’. After all, for all we know, this discovery is in the *objective* past! As a result, it is only philosophers of time, while they are discussing GBT, who use ‘the future’ to mean ‘the time beyond which nothing exists’. But this is clearly problematic, since it is *our* ordinary beliefs about the future (not philosophers’ ones) that GBT is meant to guarantee by stating that nothing exists beyond the present! It therefore seems that as soon as GBT distinguishes between two notions of the present – objective and subjective – it betrays the ordinary intuitions that initially made this theory attractive.

Another reason *why* Merrick’s suggestion is not appealing is that it makes every typical belief about the present *trivially true*, while – assuming that non-present people may also have such beliefs – we clearly think that they are wrong in holding them. Specifically, if all our typical beliefs about the present are in fact about the *subjective* present (which is to be understood as an indexical that merely refers to ‘the moment at which *these* beliefs are held or uttered’), then we cannot be wrong in holding these beliefs. People at any location in time are trivially right to believe (supposing that they do) that they are located in the present, since all that this belief requires to be true is to be held or uttered. Yet, supposing that Napoleon has the belief that the time he exists at is the present, it seems that he is wrong. Napoleon is obviously not located in the present and, therefore, he is wrong to believe (supposing that he does) that he is. In other words, not only do we think that we are right when we believe we are located at the present time, but we also think that people existing at other times (e.g., Napoleon) are wrong in holding this belief. It therefore seems that as soon as one admits that we can have the belief that we are located at the present time without being actually located at *that* time, one should allow for the possibility of being wrong in holding this belief.

A second solution to the skeptical challenge is due to Peter Forrest (2004), who claims that the epistemic objection relies on a mistaken assumption, namely that both past and present beings are *conscious* and, therefore, presumably think that the time they exist at is the objective present. According to his highly controversial ‘dead past hypothesis’, consciousness is a phenomenon that emerges only at the edge of the growing block; it is a by-product of what Forrest calls the “causal-frisson” (2004: 359). If we believe that we are located in the present then we are necessarily right to believe it, since, if we were located in the past, we would not believe anything – we would be zombies (devoid of consciousness). As Forrest puts it: “[l]ife and sentience are, I submit, activities not states. Activities only occur on the boundary of reality, while states can be in the past. […] The past is […] dead” (2004: 359). Thus, according to Forrest, Napoleon exists and he is like us in some respects (e.g., he is a physical entity just as we are), but he is not like us in all respects, as, in particular, he is not conscious. Of course, this is not to say that Napoleon has never been conscious; he was conscious when the time he exists at was the boundary of reality, since it is precisely the fact of being on the boundary of reality that gave him consciousness. However, in 2021 Napoleon is a zombie, while we are not. We can therefore be sure (by introspection) that we are located in the present (cf. also Curtis & Robson, 2016: 77–78).

Nonetheless, although Forrest’s response certainly overcomes the epistemic objection, the ‘dead past hypothesis’ brings its own range of problems. First, this hypothesis seems incompatible with GBT’s claim that there always was an edge of reality, including at times when there was no consciousness at all (e.g., 4 billion years ago): if consciousness were a by-product of the ‘causal frisson’, then consciousness should be observed *at all times*, which is obviously not the case. Of course, Forrest might reply that the ‘causal frisson’ is merely one of the many necessary conditions for consciousness to emerge. But then, it is not clear what further conditions must be met. Secondly, the ‘dead past hypothesis’ entails that presentness generates consciousness in a non-trivial sense. There are indeed many philosophers who argue that “[…] there is no interesting connection between consciousness and presentness” (Meyer, 2016: 151). Worse, there are even philosophers who defend the exact opposite view, i.e. that if there were no judgmental awareness, then there would be no presentness: “[a]n event’s occurring now depends on someone’s being judgmentally aware of it now” (Baker, 2010: 32, see also Grünbaum, 1967: 17).^6^ This is certainly not the place to settle this debate, and we do not want GBT to commit us to any controversial theory of the emergence of consciousness; so it seems preferable to reject the ‘dead past hypothesis’.

Finally, a third solution to the skeptical challenge is due to Fabrice Correia and Sven Rosenkranz (2018), who argue that the epistemic objection rests on an uncharitable rendition of the growing block view: “[…] to say, on the one hand, that the past is real (exists), and hence that so are (do) the events that once occurred, is not to say, on the other, that past events are still occurring” (2018: 89). There indeed seems that behind each formulation of the epistemic objection lies the presumption that, according to GBT, past beings (e.g., Cesar, Napoleon) still believe that they are in the objective present^7^ – which sounds absurd, since these people are long dead! This presumption clearly distorts the tensed metaphysics underpinned by GBT: the block is not like a “[…] multi-storey building, with lower floors corresponding to the more distant past, where what happens on each floor is still happening, even if it is not happening on the last floor” (2018: 89). The solution proposed by Correia and Rosenkranz is to regard ‘occurring’ as a temporary property that an event, such as ‘Napoleon is having conscious thoughts’, once had at some time earlier than now, but no longer has now (without this event having ceased to exist). It indeed seems that Broad’s original view can allow for this solution, since it is anyway committed to temporary properties (that an event once had, but no longer has), such as *being new*. Therefore, contrary to what is presumed in the epistemic objection, it does not follow from GBT that if once Napoleon believed himself to be in the objective present, he is still believing so.^8^ Assuming that there are temporary properties, it can be argued that, whereas we believe that our time is the objective present, Napoleon (who still exists in the past) once had this belief but no longer has it. In short: events can cease to occur without ceasing to exist.

However, although one may agree with Correia and Rosenkranz (and with Forrest) that past beings no longer have belief about the present (or about anything else), it is far from clear that one can always distinguish between the existence of an event and its occurrence. As Peter Geach puts it: “[o]bviously we cannot take this seriously: an actor can be distinguished from his appearance on the stage but we cannot distinguish an event on the one hand and the occurrence or emergence or appearance or taking place of the event on the other hand” (1973: 210). Of course, Correia and Rosenkranz might reply that their theory does not involve non-occurring events, but only events that do not occur at all times at which they exist. However, this reply is not satisfying. This becomes evident when considering a particular event, such as a pain (e.g., a headache): how could a pain exist at a time without being painful at that time? This sounds like a category mistake. A pain, at least as defined by the International Association for the Study of Pain, is “[…] an unpleasant sensory and emotional experience associated with actual or potential tissue damage, or described in terms of such damage” (IASP, 1994: 209).^9^ In other words, pains are *subjective*; their existence depends on feeling them. There is no time at which pains exist without being felt. Yet, Correia and Rosenkranz explicitly integrate into their ontology pains that are no longer painful. As they put it: “[one might want to] reject the idea that insofar as the past pain still exists, it still is painful, just as we reject the idea that insofar as WWI still exists, people are still dying in the trenches” (2018: 90). Thus, although Correia and Rosenkranz’s solution to the skeptical challenge rests on a fair point (GBT does not imply that events that once occurred are still occurring), it must be rejected on pain of generating paradoxical entities (such as pains that are not painful at all times they exist). Since this latter solution, like the previous two, is unsatisfying, I propose to develop in the next sections my own solution to the epistemic objection, which involves an anti-essentialist picture of kinds.

## An Anti-Essentialist Picture of Kinds

Generally speaking, it seems absurd to claim that when objects and events pass from being present to being past, they merely change with respect to their A-properties (i.e. they do not undergo any other alteration whatsoever). As Dean Zimmerman puts it: “[*e*]*verybody knows* that when events and things ‘recede into the past’ they are very different from the way they are when present” (2008: 221). It is therefore *uncharitable* to presuppose – as Zimmerman (2011), Sider (2011), Merricks (2006) and others do – that GBT *implies* that dead people are currently believing things. If it was really a consequence of GBT, there would not be a single philosopher to defend this theory! Hopefully, GBT offers room for arguing that objects and events, when they are present (i.e. on the edge of the block), are somehow different from the way they are when past. Of course, this difference could be thought as being merely *extrinsic*. For example, it might be argued that present and past things merely differ with respect to their temporal location: for objects and events, to become past is just to cease to be located *at the last time*. However, although this is a straightforward option for growing blockers, it leads to the same category mistake that was found in Correia and Rosenkranz (2018). For, to say that the difference between present and past things (e.g., present and past pains) is merely *extrinsic* is to say that things do not *intrinsically* evolve by becoming past, and therefore that they belong to the same natural kind (e.g., pains) when present and past, while past pains are not painful anymore. Indeed, since membership in a natural kind is (partly) grounded in sharing natural properties, which are usually taken to be *intrinsic* properties (cf. Bird & Tobin, 2017: § 1.1), it seems that no two entities sharing *all* their intrinsic properties can belong to different natural kinds. This is not acceptable: either a pain is painful at each time it exists, or it is not a pain. That is why it seems preferable to maintain that becoming past involves alterations in a thing’s *intrinsic* properties, to such an extent that it ceases to belong to its natural kind: a former pain is *neither* a pain that no longer exists (presentism), *nor* a pain that still exists but is not painful anymore (Correia and Rosenkranz), it is no longer a pain.

This option was considered by McTaggart himself when he wondered whether “[…] the change consisted in the fact that an event ceased to be an event […]” (1908: 459). Although McTaggart promptly rejected this option, on the ground that “[a]n event can never cease to be an event” (natural kind essentialism), it is worth reconsidering it. Natural kind essentialism is the view that membership of a natural kind is essential to its members: if *a* belongs to kind *K*, then it is an essential property of *a* that it belongs to *K* (cf. Kripke, 1980, Fine, 1994). According to this view, a pain is essentially a pain, and gold is essentially gold. Hence, a pain is always if anything a pain, and gold is always if anything gold.^10^ However, natural kind essentialism (at least as stated above) is presumably too strong. For example, in chemistry, it is readily acknowledged that “[…] a nucleus of neptunium-239 may undergo beta decay, in which one of its neutrons emits an electron leaving a proton” (Bird & Tobin, 2017: § 1.3). As a result, the nucleus in question has one more proton and, therefore, is no longer a nucleus of neptunium-239 but a nucleus of plutonium. Yet, it is intuitively the *same* nucleus (in the numerical sense of the term) that persisted through this transformation. The nucleus has thus retained its identity while undergoing a change of natural kind.^11^

Another example concerns biology, and especially Ernst Mayr’s influential biological species concept. According to this concept, species are “[…] groups of actually or potentially interbreeding natural populations, which are reproductively isolated from other such groups” (1942: 120). As Mohan Matthen (2009) points out, this definition allows for the creation of new species by the advent of reproductive isolation, which will typically split existing populations.^12^ Consequently, the existing organisms in at least one (but presumably both) of the newly isolated (sub-)populations will belong to a new species. As Matthen puts it: “[…] species membership is relational, and consequently, an organism can change its species during its lifetime […]” (2009: 95). Assuming that species are natural kinds, this case offers a second example of particulars changing their kinds. Taking the two above counterexamples seriously,^13^ it seems that nothing *a priori* precludes that objects and events may continue to exist, when no longer present, *as a different kind of entity*. For instance, ‘Napoleon is having conscious thoughts’ can be an event when it occurs in the present and be identical to something else when it is located in the past, without nothing having ceased to exist. This option has two immediate advantages: (i) it seems compatible with GBT (since it involves no annihilation) and (ii) it does not generate paradoxical entities (such as events that do not occur at certain times they exist).

Moreover, our own experience seems to match with this anti-essentialist picture. We perfectly know what some intrinsic properties are like when they are present, especially the properties we are aware of in our own conscious experience. For example, we all know what a pain, a sound or a color feels like. By contrast, a past pain, a past sound or a past color are not being felt, and so they lack this phenomenal quality. Of course, as Barry Dainton puts it, “[…] we do not know what the intrinsic character of a past experience is like, simply because as soon as an experience ceases to be present it is no longer experienced. But [he continues] we can be confident that, whatever this intrinsic character is like, it is different from that of present experiences” (2010: 20). Intuitively, the same reasoning can be applied to material things: “[…] although we know quite a lot about their causal and structural properties (e.g., the shape, size and mass of an electron […]), we have no knowledge of their non-structural intrinsic properties (e.g., what the intrinsic nature of a proton is like in itself). Given this ignorance, we certainly cannot rule out the possibility that the non-structural intrinsic properties of material things undergo changes when they become [past]” (Dainton, 2010: 20).

Taking seriously the idea that a thing undergoes an alteration in some of its intrinsic properties when it becomes past (to such an extent that it ceases to belong to its *natural kind*), the question that immediately arises is: ‘Which intrinsic property is altered?’. Timothy Williamson (2013) suggests that a thing that becomes past loses its *concreteness*.^14^ In brief, he conceives reality’s dynamic nature as grounded in temporal shifts from non-concreteness to concreteness and from concreteness to non-concreteness. According to him, a pain is thus something before and after it occurs (namely a non-concrete pain), and it is concrete only while it is occurring. Of course, this does not imply that a non-concrete pain is an abstract entity (*pace* Sider, 2001: 127). As Williamson makes clear, ‘non-concrete’ and ‘abstract’ are not to be treated as synonyms. ‘Abstract’ has its own positive paradigms, such as numbers and directions. So, when a pain disappears (and therefore becomes past), it is not transformed into an abstract object; it merely ceases to be concrete. However, although this solution may look attractive, it has (at least) two drawbacks that I mention now.

First, since Williamson provides no perspicuous account of his notion of concreteness, his proposal is not fully intelligible. As he himself concedes: “[t]he term ‘concrete’ is used informally throughout this book. For present purposes, we need not decide between various ways of making it precise (being material, being in space, being in time, having causes, having effects, …)” (2013: 6). Without any further specification, it is therefore hard to make sense of Williamson’s proposal. Secondly, assuming that non-concrete things do not occupy any spacetime region (which sounds both conventional and respectful of Williamson’s intention), it is not clear that this proposal is available to GBT. Of course, one can conceive a Williamsonian version of GBT, according to which future things do not exist, present things exist *concretely*, and past things exist *non-concretely*. However, unless the past is to be thought as an empty place, this version of GBT renders *superfluous* the existence of the past, at least understood as a physical location (i.e. a place where concrete things can be located). If the present is taken to be the unique temporal location for concrete things, then there is no reason to assume that other temporal locations (such as the past) exist – these temporal locations would always remain empty, after all. It therefore seems that if one adopts the Williamsonian version of GBT, one must reject the view that some things can be temporally located elsewhere than in the present. This makes this version of GBT (i) very similar to certain versions of presentism, such as ‘thisness presentism’ (according to which past things survived by abstract entities: their thisnesses)^15^, and (ii) difficult to reconcile with physics (according to which concrete things can be located elsewhere than *now*, just as they can be located elsewhere than *here*).

A Williamsonian reply could be that, although Socrates is not in space *now*, i.e. Socrates is not located at the present three-dimensional slice of the four-dimensional manifold (this is presumably the sense in which Socrates is non-concrete *now*), he *does* occupy a spacetime region *now*, in the sense that he *was* in space some years ago. In this regard, the spacetime region that Socrates occupies *now* is, for instance, wholly before the spacetime region that Napoleon occupies *now*. However, although one must acknowledge that ‘being in space’ (unlike ‘being in spacetime’) can be introduced as a relative notion and, therefore, that a thing can be located in spacetime *now* without being located in space *now*, this reply misses its target. The reason is that this should not be the sense in which growing blockers say that, for instance, Socrates *is* in spacetime. I mention two arguments in favor of this claim. First, even presentists (who deny the existence Socrates) would agree that Socrates *was* in space some years ago and, therefore, that he is located in spacetime *now* (in the above sense). Yet, since GBT and presentism crucially disagree on what is located in spacetime *now* (e.g. either both Socrates and Obama, or only Obama), it seems that ‘being in spacetime’ should have a more specific meaning within GBT, which entails that everything that is in spacetime *now* is also in space *now*. Secondly, supposing that a thing can be in spacetime *now* without being in space *now* (as permitted by the above sense of ‘being in spacetime’), it follows that the hundredth President of the United States (who does not exist according to GBT) does occupy a spacetime region *now*, simply because he *will be* in space in a few years. This is not acceptable. Growing blockers should therefore argue that everything that is located in spacetime *now* is located is space *now*, and hence that Socrates is still (and will always be) a concrete entity (*pace* Williamson).

So, if ‘concreteness’ is not the right answer to provide to the question ‘Which intrinsic property is altered when a thing becomes past?’, what could it be? As a first approach, the most plausible answer is ‘it depends’: the intrinsic properties concerned by the alteration depend upon what kind of thing is becoming past.^16^ There are indeed good reasons to believe that a person, a pain, and a stone do not change in the same way by becoming past, since they did not share the same bedrock of intrinsic properties in the first place. For example, while a person who dies (and therefore becomes past) presumably loses the property of being conscious, it would be absurd to extend this to all entities, since most of them have never been endowed with consciousness. Likewise, events seem to no longer occur when past, while this cannot be said about people or stones.

For now, all that can be said in order not to fall back into the pitfalls of Correia and Rosenkranz’s theory is that whatever the sort of alteration a thing that becomes past undergoes, this implies that this thing no longer belongs to its *natural kind* (rejection of natural kind essentialism). A person that is no longer conscious is no longer a person; an event that no longer occurs is no longer an event; and a pain that is no longer painful is no longer a pain.^17^ Yet, just as it is the same nucleus that persists through the addition of a proton to become a nucleus of plutonium, these are the same things that persist through the passage of time to become past. There is therefore nothing such as ceasing to exist; when past, the same things continue to exist, falling under a different natural kind. Given this, the interesting question to ask is not ‘Which property is altered when a thing becomes past?’ (since the answer varies depending on what kind of thing is considered),^18^ but rather ‘What remains of a thing that became past?’. For example, ‘What remains in 2022 of Napoleon’s belief that he is located in the present?’. In order to answer this question, I will introduce, in the next section, the notion of ‘bare particular’. This notion will be conceived, in a deliberately open-ended manner, as what is responsible for the continuity of existence of both continuants (people, tables, planets, etc.) and occurrents (events, processes, etc.), through both superficial change (e.g., becoming warm) and radical change (e.g., becoming past).

## The Virtues of Bareness

If one wants to account for how things located in the past exist, it is worth considering the primitive notion of a ‘bare particular’. This notion comes from the substratum theory, according to which “[…] particulars are, in a certain sense, separate from their universals” (Sider, 2006: 387). More specifically, the substratum theory says that particulars and universals are only connected to each other by a relation of instantiation. That means that particulars do not have properties *as parts*; they instantiate them. As Ted Sider puts it: “[t]hey are nothing but a pincushion into which universals may be poked” (2006: 387). John Locke speaks of them as the “I know not what” substrata (1689, II, xxiii, § 2), while Plato (2000) uses the term “receptacles” (*Timaeus* 48c-53c).^19^ I personally prefer the expressions ‘bare particular’, ‘substratum’, or ‘*thin* particular’ (as opposed to ‘*thick* particular’ which refers to the fusion of a *thin* particular and its universals).^20^ In other words, a ‘bare (or *thin*) particular’ is the mereological difference between a thick particular and its universals. Whereas bare particulars play a predominant role in the individuation debate – they are usually posited to account for the identity and distinctness of particulars (cf. Bergmann, 1967; Moreland, 2001; Sider, 2006) – they might also, as we will see, be helpful in the philosophy of time.

Importing a notion issued from the individuation debate into the philosophy of time is less crazy than it might seem. After all, in the *Physics* (Book I, Chap. 6), Aristotle (1999) himself connects the notion of a ‘material substratum’ (in which the properties exemplified by a particular inhere) to the question of change. He argues that any change must be analyzed in reference to an invariant substratum. However, it would be a mistake to claim that the notion of a ‘bare particular’ has an antecedent in Aristotle; for, he explicitly says that everything that exists (with the notable exception of the eternal substance of the unmoved mover) is a compound of matter and form (hylomorphism). Specifically, Aristotle distinguishes two types of change: sometimes a change is just a change in a characteristic or two, as when the cold bronze sphere becomes a warm bronze cube, and sometimes the matter itself changes and we are no longer dealing with bronze at all (cf. Cohen, 1996: 67–68). The first type of change is called ‘reciprocal’, since there can be a transformation back into the original stuff, whereas the second change is called ‘nonreciprocal’, since it is definitive. However, both types of change require something that survives the transformation (otherwise, the process would not be called ‘a change’), namely a substratum which is responsible for the continuity of existence – this substratum is matter (*hyle*), which is not to be understood as a bare particular, but as a pure potentiality. As Christopher Byrne explains: “[t]hese requirements apply to essential change just as much as to accidental change, for Aristotle insists that there is a persisting substratum in generation and destruction as well” (2018: 47). Of course, it is the second type of change (i.e. ‘nonreciprocal change’) that will be matter of great concern in the remainder of this chapter, since becoming past is, in every sense of the expression, a definitive process.

Against the substratum theory there is the bundle theory, according to which “[…] particulars are just bundles of universals” (Sider, 2006: 387).^21^ Although substratum and bundle theorists agree on much – e.g., they agree that both particulars and universals exist, and that a particular somehow *has* universals – they do not share the same conception of what a particular is. Whereas a bundle theorist affirms that a particular is nothing but all the universals it has (i.e. it is the mereological fusion of all its universals), a substratum theorist denies this. According to the latter, when you take a particular, and you mereologically subtract away all its universals, there is something left: a bare particular. So, assuming the principle of uniqueness of mereological fusion (no universals can have two fusions), the bundle theory and the substratum theory differ sharply over the possibility of exactly similar particulars: whereas a bundle theorist affirms that no two particulars can have exactly the same universals (since a particular is just the sum of its universals),^22^ a substratum theorist affirms that distinct particulars *can* have exactly the same universals (since they will have distinct bare particulars, i.e. distinct non-universal ‘cores’). The main benefit of adopting a substratum theory rather than a bundle theory is that only the former avoids getting committed to the controversial Principle of Identity of Indiscernibles (PII), according to which necessarily, any two particulars that have all the same qualitative properties are the same particular. There indeed seems to be many classes of particulars for which PII fails to apply (cf. Black, 1952: 153–164, French, 2015: §§ 2–4).

Now, assuming that bare particulars constitute a fundamental ontological category, it might be argued that, although *becoming past* involves alterations in a thing’s intrinsic properties (to such an extent that it ceases to belong to its *natural kind*), the bare particular of *that* thing will continue to exist. For example, if the Battle of Waterloo is conceived as a temporally-extended particular instantiating some properties^23^ (e.g., having opposed France to the Seventh Coalition, having been won by the Duke of Wellington), then this battle will always be something when no longer occurring: a bare particular. This bare particular is what persisted through the intrinsic alteration that affected the battle of Waterloo when it became past. As an analogy, consider the case of a statue made of bronze, and suppose, for the sake of argument, that bronze is the substratum. The statue is, in that sense, bronze instantiating some properties (e.g., *having the shape of a woman*, *being cast by Rodin*). Now, suppose that this statue is melted so that all that remains is a warm bronze cube. Clearly, after such a process, bronze has lost some properties (e.g., it no longer has the property of *having the shape of a woman*, nor the property of *being cast by Rodin*). As a result, it can no longer be called ‘a statue’ (or perhaps only in a non-trivial sense by a bunch of contemporary artists). Nonetheless, bronze survived the transformation. Of course, this analogy has limits: whereas melting is a superficial (or reciprocal) change which does not affect bronze in its proper kind (bronze remains bronze), becoming past is a radical (or non-reciprocal) change which definitely turns every thick particular into a bare particular.

To the question ‘What remains of a thing that became past?’ a plausible answer might therefore be: a bare particular. An immediate objection is that, according to this answer, the proper name ‘Socrates’ seems to refer to two distinct entities: a thick particular ‘T’, which has gone out of existence, and a bare particular ‘B’, which is still with us. This poses at least two issues: (i) it is not clear whether Socrates should be identified with B, T or both B and T, and (ii) it seems that T’s going out of existence is incompatible with GBT. Let us start with the first issue. At first sight, it seems perfectly acceptable and convenient to say that Socrates *is* T, and that Socrates *is* B. The most natural view is indeed that Socrates came into existence in 469 BC, then lived as a thick particular until he drank the hemlock in 399 BC, and continues to exist as a bare particular since then. To put it another way, just as it seems that it is the *same* bronze that is a statue at *t*_1_ and a warm cube at *t*_2_, it seems that it is the *same* particular (Socrates) that is thick in 350 BC, and bare in 2021 AD. This natural view escapes, by the way, the second issue, since it does not imply any form of annihilation: there is simply a single entity (Socrates) which, although it has existed in two different forms (a *thick* particular and a *bare* particular), has never (and will never) cease to exist.

Unfortunately, things are not so simple, especially because if Socrates *is* both B and T, then it follows that B *is* T, which seems to betray Leibniz’s Law. To illustrate, suppose that *t* is a moment at which Socrates was alive: at *t*, T has the property of *being alive* as a part, but at *t*, B does not have the property of *being alive* as a part. By the Principle of the Indiscernibility of Identicals (necessarily, if two particulars are identical, then they have all the same qualitative properties), it follows that B is not identical to T. This is analogous to a well-known situation in the metaphysics of constitution, which is usually introduced as ‘the puzzle of Tibbles the cat’ (cf. Wiggins, 1968, Geach, 1980, Burke, 1996). In brief, suppose that before us stands a cat named ‘Tibbles’. Before us is also that part of Tibbles which consists of all of Tibbles except his tail. Let us call that part of Tibbles ‘Tib’. Since Tibbles has a tail, but Tib does not, it follows, by the Leibniz Law, that Tib is not identical to Tibbles. Suppose now that Tibbles loses his tail. At this moment, we are inclined to say that Tib is identical to Tibbles. After all, Tib and Tibbles now occupy the same volume at the same time and, as David Wiggins puts it, “[i]t is a truism frequently called in evidence and confidently relied upon in philosophy that two things cannot be in the same place at the same time” (1968: 90). Hence, assuming that identity is not contingent, we are faced with a contradiction: Tib *is* and *is not* identical to Tibbles. Of course, claiming that either Tib or Tibbles has ceased to exist is not an option for growing blockers and, in any case, would be arbitrary. As Michael Burke makes clear: “[t]he identity of a cat surely is not tied to its tail. So Tibbles still exists. But surely Tib has not ceased to exist: Tib lost none of its parts” (1996: 63). So, what should we do?

First, it must be noted that the puzzle of Tibbles the cat does not only concern bare particularism, but any view that analyzes change in reference to an invariant substratum and, thereby, allows things to retain their identity while losing parts. Therefore, if one wants to reject bare particularism on this basis, one should be ready to endorse a view such as mereological essentialism, i.e. “[…] the doctrine that every part of an object, no matter how small, is essential to its identity” (Burke, 1996: 63), which brings its own range of problems. For example, how can mereological essentialism account for the fact that common-sense objects persist through change? Considering that bananas ripen and houses deteriorate, how can this view say that they are the same things, if they are not quite the same? Secondly, there is a battery of well-known solutions to the puzzle of Tibbles the cat, which strongly suggests that the above objection against bare particularism is not definitive. For example, some philosophers opt for four-dimensional entities whose parts may extend in time as well as in space (cf. Heller, 1990, Lewis, 1986, Sider, 2001), some others argue that identity is a contingent relation that may hold at some times but not at others (Gibbard, 1975, Myro, 1985, Gallois, 1990), Peter van Inwagen (1981) claims that there are no such things as arbitrary undetached parts, David Wiggins (1968) distinguishes between the ‘is’ of constitution and the ‘is’ of identity, etc. The question is then, ‘Which of these solutions are available to bare particularism within GBT?’ It would be difficult to provide an exhaustive answer to this question, but it *a priori* seems that at least two solutions could be retained: (a) relativizing numerical identity, and (b) distinguishing between two senses of ‘is’ (constitution and identity).

However, option (a), which amounts to saying that B was identical to T when Socrates was alive and that B is now distinct from T since Socrates died, has two major drawbacks: (i) it looks *ad hoc* (at least in the present context), and (ii) it forces one to reject the ‘constituent thesis’, according to which every thick particular has a bare particular (and some properties) as constituents, which is at the core of bare particularism (cf. Bailey, 2012). Of course, this is not to say that these drawbacks cannot be overcome. Niall Connolly (2015), for example, happily rejects the ‘constituent thesis’. He argues that the best version of bare particularism takes the relation between a substance (e.g., a tree) and its substratum to be *identity*, and not the relation of constitution. Nonetheless, a better option (which requires fewer concessions) is to preserve the ‘constituent thesis’, by arguing that when one says that Socrates is T and that Socrates is B (which again is perfectly acceptable and convenient), one actually means that Socrates *constitutes* T and that Socrates *is identical to* B. The distinction between these two senses of ‘is’ was first highlighted by David Wiggins (1968) and Peter Geach (1980). As, for instance, Wiggins puts it: “[t]he ‘is’ of material constitution is not the ‘is’ of identity. The tree is *made of* (or *constituted of* or *consists of*) *W*, but it is not identical with *W*. And ‘*A* is something over and above *B*’ denies ‘*A* is (wholly composed of) *B*’ or ‘*A* is merely (or merely consists of) *B*.’ If *A* is something over and above *B*, then of course *A* $\ne$ *B*, but the proper point of saying ‘over and above’ is to make the further denial that *B* fully *exhausts* the matter of *A*” (1968: 91–92). It is however worth noting that, according to Wiggins’ constitution view, the substratum *W* which constitutes the substance *T* is not to be understood as a bare particular, i.e. as something ‘over and above’ *T*, but as a ‘superinternal relation’^24^ that organizes the parts of *T*. So conceived, the relation of constitution is distinguished from identity insofar as it is asymmetric: *W* constitutes *T*, but not vice versa.

**Fig. 1:**
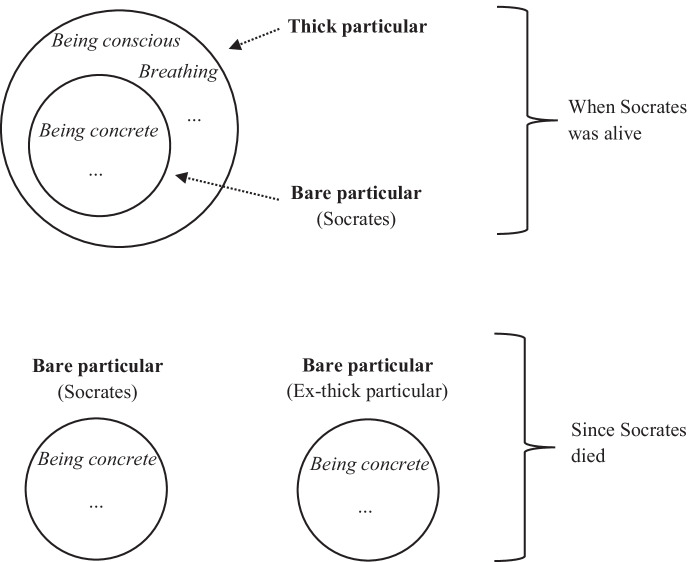
Bare and thick particulars at two different times

Now that we have distinguished between the two senses of the word ‘is’ (constitution and identity), we can return to the case of Socrates and see how this applies to it. As a reminder, GBT commits us to the claim that T still exists now, just like B. A question then arises: ‘What are the properties that T now has?’. Certainly not those that were specific to Socrates when he was alive (e.g., being *conscious*, *breathing*, etc.). The most plausible answer is that T and B now have exactly the same properties. Taking this seriously, the situation is as follows: B has always been a bare particular (at least since it began to exist); on the other hand, T is bare now but has not always been so: when Socrates was alive, T was a whole constituted by (i) a bare particular (namely B) and (ii) properties such as *breathing* and *being conscious*. The situation is depicted in Fig. 1. An immediate objection is that this version of bare particularism is ontologically costly: it generates a colossal number of bare particulars. A reply could be that, from a qualitative point of view, the doctrine remains parsimonious, by keeping down the number of *sorts* of entities (it only involves thick and thin particulars). And, according to Lewis (1973: 87), it is the only parsimony criterion one should consider.

Another objection is formulated by Andrew Bailey (2012). Roughly, this objection says that if one accepts ‘the constituent thesis’ (i.e. the thesis that every thick particular has two kinds of constituents: its properties and its bare particular), then one should abandon ‘the having thesis’ (i.e. the thesis that every thick particular has its properties by having as constituents properties that are instantiated by another of its constituents: its bare particular). Proof is that, taken together, these two theses imply that two co-located entities (the bare particular and its host thick particular) have the same properties, which sounds false. However, although persuasive, this objection exerts no pressure on the above view, since ‘the having thesis’ is not taken to be true. In general, a bare particular does not instantiate the properties had by its host thick particular (rejection of ‘the having thesis’); it merely has these properties in a derivative way. For example, Socrates only had in a derivative way the properties that one usually attributed to him (e.g., *being conscious*, *breathing*, etc.). Does this undermine the view as a version of bare particularism? It does not seem so, since other self-appointed ‘bare particular theorists’ reject ‘the having thesis’ (cf. Wildman, 2015).

The claim that what remains of a thick particular that became past is a bare particular looks attractive for the following four reasons. First, the notion of a bare particular is (at least in itself) *economical*: it is an ontological free lunch for those who accept the classical substratum theory of individuation, according to which particulars are not exhausted by the properties they exemplify, but are associated with a substratum. Secondly, the notion of a bare particular is *familiar*: it echoes a long-standing conception of change, according to which change is possible only if something always persists through any change that occurs. In this perspective, ‘becoming past’ should just be conceived as a radical (or nonreciprocal) type of change. Thirdly, this view is *compatible* with GBT’s main imperatives: the possibility that thick particulars may continue to exist as a different kind of entity (a bare particular) is not excluded by the ontological preservation of what is no longer present, which is an essential feature of GBT. Fourthly, bare particulars have a great *explanatory power*: they allow for a conception of the past – ‘the bare past’ – in which nothing occurs (e.g., there are no events, no movements, so that no property can be gained or lost) and, therefore, offer an appealing account for the fixity of the past. For all these reasons, it seems that bare particulars should be conceived as what is left of a thick particular when certain of its intrinsic properties were subtracted by the passage of time. Quentin Smith endorses a similar view when he claims that “[past particulars] are ‘bare particulars’ in the sense that they lack nonrelational, monadic properties” (2002: 132). But while Smith’s view (so-called ‘degree presentism’) attributes this ‘bareness’ to the fact that past particulars are only partly real,^25^ GBT should not allow for degrees of existence: past and present particulars are just as real.

Unfortunately, as previously said, it seems difficult to identify one unique intrinsic property whose loss would make particulars turn from thick to bare, since thick particulars of various kinds (e.g., events, people, stones) do not share the same bedrock of intrinsic properties in the first place. Nonetheless, it seems possible to provide an informative criterion: the properties that a particular loses when it turns from thick to bare are (at least) the ones that make it belong to the natural kind to which it belongs when present. These are the properties in terms of which natural kinds are traditionally defined. For example, in June 1815, if it is the property of *occurring* that makes a thick particular, constituted by the battle of Waterloo, belong to the kind ‘events’ (Simons 2003: 357), then it is at least this property that the particular in question loses when it becomes past. Likewise, in the fifth century BC, if it this the property of *being a featherless biped* (*Categories*, 3a 23 − 5), or the property of *being a rational animal* (*Politics*, 1253a 10), that make a thick particular, constituted by Socrates, belong to the kind ‘man’, then these are at least these properties that the particular in question loses when it becomes past. In other words, ex-thick particulars are free from all the properties that jointly define the natural kind to which they belonged when present. This is what turns them to bare. This criterion does justice to the idea that both continuants and occurrents continue to *concretely* exist when no longer present, but as a different kind of entity (rejection of natural kind essentialism) – a bare particular – which results from the alteration of some specific intrinsic properties they possess.

It is important to stress that bare particulars are *individuals*, not properties, and are *concrete* (i.e. they occupy spacetime regions), not abstract *entities*. Consequently, they cannot be confused with *thisnesses*, where the thisness of a thing *x* is the abstract non-qualitative property of being *x* (or the abstract non-qualitative property of being identical to *x*).^26^ In this sense, the thisness of a thing *x* is *not* merely a conjunction of all of *x*’s qualitative properties, but it is *x*’s property of being just *that* thing. For example, the thisness of an event, such as the Battle of Waterloo, is the property of that particular event, and of nothing else. The reason *why* it is worth distinguishing bare particulars from non-qualitative thisnesses is that both of these notions stem from the individuation debate: they are posited as competitive alternatives to the view according to which particulars are just bundles of universals. More specifically, substratum and thisness theorists agree that there may be distinct particulars that do not differ in respect of the universals they possess. But, whereas the former thinks that a particular is individuated by its substratum, the latter thinks that it is individuated by its thisness.

Of course, the nature of thisness is controversial, but one view is that thisnesses are primitive, i.e. they are elements of reality that cannot be reduced to anything more fundamental. Those who believe in primitive thisnesses typically believe that a thisness comes into existence with its thing, and continues to exist as long as it is exemplified by *that* thing. But some philosophers, e.g., Robert Adams (1986), Simon Keller (2004), and David Ingram (2016, 2018), go a step further by arguing that the property of thisness continues to exist, even though it is no longer exemplified. As Ingram puts it: “[o]n my view, for any entity x, x’s thisness *T* comes into being with x, *T* is uniquely instantiated by x throughout x’s existence, and *T* continues to exist uninstantiated when x ceased to exist” (2018: 61). Following these people, it might therefore be argued that non-instantiated thisnesses – rather than bare particulars – are what is left of things (e.g., events) when they become past.

However, this option does not seem available to GBT. The most obvious reason is that friends of the thisness view hold that past entities cease to exist while being survived by their thisnesses, which is incompatible with GBT’s claim that nothing ever ceases to exist (Broad, 1923: 66). Moreover, just as with Williamson’s proposal, the ‘thisness view’ renders the existence of the past *superfluous*. As has been said, thisnesses (and therefore non-instantiated thisnesses) are *abstract entities*, while abstract entities are generally taken to occupy no spatiotemporal regions. Thus, unless the past is to be thought as an empty place (i.e. a place where no concrete entities are located), the ‘thisness view’ can only be accommodated by presentism, which takes the present to be the unique temporal location for concrete entities. It is no coincidence that most philosophers accepting thisness ontology – Adams (1986), Keller (2004), Ingram (2016, 2018) – are presentists.

Of course, as Ted Sider has observed, there is a complaint against bare particulars: “[they] are widely regarded as the grossest of metaphysical errors” (2006: 392). For example, it might seem that if a thing has no properties, then there is at least one property that this thing has, namely the property of having no properties, which renders the notion of ‘bare particular’ incoherent. An immediate reply is that if the objection is that bare particulars have no properties at all, then the objection is just wrong. Once again, the bare particulars involved in the above view of ‘how things become past’ do not need to be free from all properties, but merely from those that together make *these* things belong to the natural kind to which they belong when present. In that sense, bare particulars do have some properties, such as *being a particular*, *being bare* or *being concrete* – the nature of a bare particular is, by the way, given by the properties it instantiates.^27^ In that respect, the expression ‘bare particulars’ is misleading; it does not mean that some particulars are entirely free from properties, but rather that properties are not constitutive parts of a substratum. As Bradley Rettler and Andrew Bailey make it clear: “[b]are particulars are ‘bare’ in at least this sense: unlike objects, they have no properties as parts” (2017: § 3.2). In other words, the expression ‘bare particulars’ conveys the idea that the link between a substratum and the properties it bears cannot be a relation of constitution, but must be a relation of instantiation; otherwise the substratum (assuming it has no further constituents) would be just a bundle of properties.

A more charitable interpretation of the objection, then, is that if particulars were wholly distinct from their universals, it would be possible for there to exist *truly* bare particulars (i.e. particulars that instantiate no properties at all), while this proposal is incoherent. Again, *truly* bare particulars would have at least one property, viz. the property of having no properties. In reply, three things can be said. First, one can prevent the possibility of *truly* bare particulars through an appropriate conception of modality (though this does not seem desirable).^28^ David Armstrong (1989), for instance, builds the impossibility of *truly* bare particulars into his theory of possibility. Secondly, a similar objection can be addressed to bundle theorists, since nothing *a priori* excludes the possibility “[…] where no universal is compresent with any universal, not even itself” (Sider, 2006: 392). Thirdly, and perhaps most importantly, this objection rests on a confusion between *sparse* and *abundant* properties.^29^ In the abundant sense of ‘property’, each meaningful predicate corresponds to a property; so “[…] if we could predicate ‘has no properties’ of a thing, then that thing would indeed have a property corresponding to the predicate” (Sider, 2006: 392). But this is clearly not the relevant sense here; rather ‘property’ is to be understood in the sparse sense: just as *being red* or *being red or round*, *has no property* does not correspond to a property. As Sider puts it: “[j]ust as a thing can be red or round without having a sparse property of being red or round […], a thing can have no sparse properties without having a property of having no sparse properties. And of course, the substratum theorist’s universals are sparse” (2006: 392).

A further complaint might be that substratum theorists are unable to give a coherent account of the instantiation of universals, which is a major reason for rejecting their theory. As David Lewis puts it: “[c]onsider the predicate ‘instantiates’ (or ‘has’), as in ‘particular *a* instantiates universal *F*’ or ‘this electron has a unit charge’. No one-off analysis applies to this specific predicate” (1983: 353–354). However, although one must acknowledge that most substratum theorists remain silent on the predicate ‘instantiates’,^30^ it is not clear that such an analysis has to be provided. In particular, it seems that substratum theorists can argue that ‘instantiates’ is part of their ideology – this might even seem required, since postulating a dyadic universal of instantiation (to bind particulars to their universals) would at best postpone the need for primitive predication and, therefore, generate an uneconomical regress. Moreover, it is not clear that bundle theorists are in a better position: they need to take the predicate of ‘compresence’, i.e. the predicate that relates the universals had by a given particular to one another, as primitive. This indeed seems required in order (i) to say which fusions of universals count as particulars (e.g., there are fusions containing the universals of goldenness and mountainhood as parts, whereas there is no particular such as a golden mountain),^31^ and (ii) to prevent that any universal had by a part of a particular is had by that particular (e.g., Geneva is a town, while Switzerland is not a town, though Switzerland has Geneva as a part).^32^

A last complain concerns proper names. Against the descriptivist tradition (cf. Frege, 1982), names are typically seen as having no linguistic meaning beyond their reference. Ruth Barcan Marcus (1961), for instance, argues that proper names should be regarded as ‘tags’, since they refer *directly* to their bearers, i.e. not by way of descriptions. Taking that for granted, the question that arises is: “[…] what it is, if not an associated description, that fixes what a name refers to [?]” (Reimer, 2019: § 2.2). The most popular answer to this question is the so-called ‘causal theory of reference’, according to which (i) a name’s referent is fixed by an original act of naming, and (ii) subsequent uses of that name succeed in referring to that referent by being linked to the original act of naming via a causal chain (cf. Kripke, 1980). As Marga Reimer puts it: “[…] speakers thus effectively ‘borrow’ their reference from speakers earlier in the chain, though borrowers needn’t be able to identify any of the lenders they are in fact relying on” (2019: § 2.2). This popular answer, however, might seem at odds with bare particularism. Suppose that the proper name ‘Socrates’ refers to a bare particular. Since bare particulars are typically taken to be causally powerless (causal powers are necessarily connected with natural properties, after all),^33^ it might seem that the link between ‘Socrates’ and its referent is broken. As Keith Campbell puts it: “[a]ll causal action is exerted by way of the properties of things and all effects are effects on the properties of things. The substratum, precisely because it is without properties, including passive powers, ought to be totally immune to all causal activity” (1990: 9). In reply, one should insist on the fact that bare particulars being powerless does not imply that the reference-link is broken.^34^ For instance, although Socrates is a bare particular, and therefore is powerless (after having been causally active, through a thick particular he constituted, for more than seventy years), we can still successfully refer to him. The reason is that *our act of referring* is causally linked to the original naming of Socrates (by which ‘Socrates’ became a rigid designator of that particular). Specifically, Socrates (via his initial baptism) is at the origin of a causal chain (which ensures that later uses of the name ‘Socrates’ succeed in referring to him), although he is devoid of all the properties in virtue of which he was (in a derivative way) causally active.

At the end of the day, the continued existence of bare particulars seems to offer an elegant story (i.e. a story that does not generate any paradoxical entity) about the kind of change continuants (people, tables, planets, etc.) and occurrents (events, processes, etc.) undergo when they become past. In particular, once an event ceases to be present, it is no longer in any sense occurring and, therefore, it is no longer in any sense an event (rejection of natural kind-essentialism). Of course, this is not to say that something ceases to exist; what remains (and will always remain) from *that* event is a bare particular, i.e. a substratum that is at least freed from the property of *occurring* (supposing that this property is what makes a particular belong to the kind ‘events’). Events are thus to be thought as a natural kind to which some particulars do belong when present; by becoming past, the same particulars continue to exist but, since they have undergone an intrinsic alteration (which involves the loss of the property of *occurring*), they are now bare. Is this story sufficient to solve the epistemic objection? The answer is ‘not quite’. It cannot be simply because my believing is occurring that I know that this belief is located at the present time; I might well ignore that it is occurring, in which case I would not know that it is located at the present time. What is further required to solve the epistemic objection is the claim that, as a matter of general fact, if one’s belief is occurring, then one knows it by introspection. Thus, the reason *why* we know that our time is the objective present is that (i) such an event (i.e. ‘someone’s believing to be present’) could not occur in the past (which is to be conceived as a temporal region exclusively populated by bare particulars), and (ii) we introspectively know that our belief that we are located in the present is occurring.

**Fig. 2:**
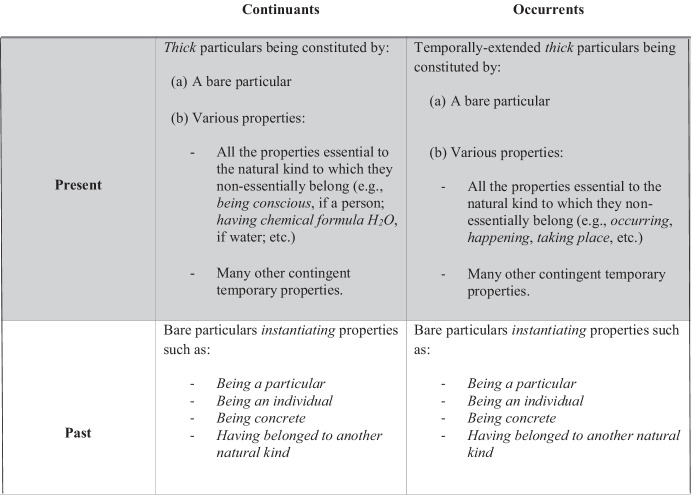
Continuants and occurrents at present and past times

To be sure, the above conception of the past – ‘the bare past’ – allows growing blockers to deny that GBT entails that events are still occurring in the past (*pace* Bourne, 2002, Braddon-Mitchell, 2004, Merricks, 2006). Indeed, assuming that bare particulars are what will forever be left of events, GBT coheres with the intuitive idea that an event, such as ‘someone’s believing to be present’, can only occur at the present time. Accordingly, ‘Napoleon’s thinking about crowning the Emperor in the present’ is *not* occurring in the past; rather, ‘Napoleon’s thinking’ occurred, and what remains of that former event is a bare particular. Then, given that if one’s belief is occurring, one introspectively knows it, we can be confident that we are located in the present, i.e. at the leading edge of the growing block, when we think we are (rejection of skepticism). Thus, contrary to what the epistemic objection states, we (as constituents of conscious events) do find ourselves in a far better epistemic position than Napoleon, who is no longer the constituent of any event whatsoever. The situation is summarized in Fig. 2.

Does ‘the bare past’ undermine the intuitiveness of GBT, while growing blockers typically take intuitiveness as a reason to endorse their metaphysical view of time? I think that the answer is no. I acknowledge that bare particulars might appear as exotic entities, although I suspect that this has more to do with the term ‘bare particular’ than with the concept itself. After all, this notion comes from the substratum theory, which is arguably the most intuitive theory of individuation; at least, the idea that particulars are, in a certain sense, separate from their universals, seems to be widely shared. Likewise, with respect to the question of change, the idea that for something to change, something (that is responsible for the continuity of existence), thus potentially a bare particular, must survive that change, also seems intuitive. But, let us leave that aside. The important point is that, even if bare particulars should definitely be regarded as exotic entities, this would not detract from the intuitive character of GBT. To be clear, a metaphysical theory of time, such as GBT, is not intuitive in the sense that it resorts only to intuitive entities – this would be an unreasonable requirement for a theory that aims at ruling on the structure of reality. Rather, a metaphysical theory of time is intuitive in the sense that it can account for some basic intuitions we have regarding the nature of time. In that respect, positing bare particulars allows GBT to account for (at least) two intuitive aspects of reality: (i) the fact that existing in the present differs from existing in the past (e.g., Napoleon, unlike us, is no longer conscious; he is long dead), and (ii) the fact that the past is fixed (e.g., since nothing happens in the past, there is nothing we can do to change it). The label ‘intuitive theory’ of GBT seems therefore to be secured.^35^

## Conclusions

Despite some attractive features, GBT is often criticized for not being a *viable* alternative to presentism because of the ‘epistemic objection’. According to this objection, GBT would provide no basis for saying that our time is the objective present. Worse, this theory would commit us to conclude that we are, almost certainly, located in the objective past. However, as has been argued, this objection relies on a mistaken assumption, namely that the reality of the past entails that events are occurring in the past. Indeed, it is one thing to say that the past is real, it another to say that past events are still occurring. In particular, assuming that bare particulars of past events will always exist, GBT can be reconciled with the intuitive idea that events can only occur at the present time. In this perspective, becoming past involves alterations in a thing’s intrinsic properties to such an extent that it ceases to belong to its *natural kind*, while the bare particular of *that* thing will continue to exist. The intrinsic properties that are lost in the process are (at least) those that define the natural kind to which the thing in question belonged when present. As a result, since (i) there are no events – such as ‘Napoleon’s thinking about crowning the Emperor in the present’ – occurring in the past, and (ii) if one’s belief is occurring, then one introspectively knows it, we (as constituents of conscious events) can be confident we are located in the present, i.e. at the leading edge of the growing block.

## Footnotes

1 For instance, GBT seems ontologically well designed for accounting for the intuitive asymmetry between the ‘open future’ and the ‘fixed past’ (cf. Grandjean 2021a, b, Correia & Rosenkranz, 2018).

2 B-theories of time are not affected by the epistemic objection, due to the indexical nature of “present” on these accounts (cf. Cameron, 2015: 8).

3 It should be remembered that on December 2, 1804 Napoleon crowned himself.

4 Nonetheless, Cameron (2015: 23) argues that the epistemic objection faces *every* A-theory of time, not only the non-presentist ones. According to him, appealing to the fact that presentism entails that we are now in the objective present does not help to solve the epistemic problem of *how* we know that we are in the objective present (unless we can come to know that presentism is true, which seems just as difficult as knowing that we are now in the objective present). Cameron’s solution to the epistemic objection, which is available to every A-theorist of time, is to adopt an externalist account of knowledge according to which we do not have to have any subjective evidence that the time we exist at is the objective present in order to know that it is so (cf. Cameron, 2015: 49–50). Of course, the main weakness of Cameron’s solution is that it crucially depends upon whether externalism is a viable theory of knowledge, while many epistemologists think it is not.

5 Growing blockers must also distinguish the ‘*objective* past’ (i.e. the past that precedes the *objective* present) from the ‘*subjective* past’ (i.e. the past that precedes the *subjective* present) (cf. Merricks, 2006: 106).

6 This does not necessarily imply that the existence of time itself requires consciousness. Barker (2010: 32) argues even without conscious observers, there would still be instants of time that are ordered by the earlier-than relation (cf. also Meyer, 2016, pp. 145–147).

7 Correia and Rosenkranz (2018: 86) acknowledge that the presumption that lies behind some formulations of the ‘epistemic objection’ (e.g., Bourne, 2002: 362) might be less radical: while past beings no longer believe to be in the objective present at that time, their beliefs are nonetheless answerable to how things stand at that time. However, this second presumption also fails: what needs to be taken into account in order to evaluate past beings’ beliefs is only how reality was back then. In particular, the fact that reality has since then grown is irrelevant for the assessment of past beings’ beliefs (cf. Correia & Rosenkranz, 2018: 94).

8 Nonetheless, as Correia and Rosenkranz argue, not only does Napoleon still exist, but so does his belief. However, this does not imply that Napoleon’s belief to be in the objective present is false *now*: beliefs of tensed propositions do not change their alethic status depending on the changes in worldly conditions (cf. Evans, 1985: 349–350). In this sense, the fact that, back then, reality was such that Napoleon’s time was the last time (i.e. the edge of the block) is the only one relevant for the assessment of Napoleon’s belief to be in the objective present. Therefore, there is no sense at all in which Napoleon is now mistaken: if Napoleon’s belief was knowledge at the time this belief was entertained, it still is a piece of knowledge now (cf. Correia & Rosenkranz, 2018: 92).

9 I take the example of ‘pains’ because it is, in my opinion, the most striking one. But there are good reasons to think that this objection can be extended to any type of event (including non-subjective ones), since an event is generally defined as “[…] anything that happens, takes place, or occurs” (Simons, 2003: 357).

10 It must be acknowledged that the move from ‘*x* is essentially *F*’ to ‘*x* is *F* whenever it exists’ is substantial. Proponents of natural kind essentialism might therefore be more inclined to move from ‘*x* is essentially *F*’ to ‘*x* is necessarily *F* if it exists’.

11 As Bird and Tobin (2017: § 1.3) have made clear, this example is consistent with the thesis that it is essential to neptunium that its nuclei have 93 protons whereas it is essential to plutonium that its nuclei have 94 protons. In other words, the rejection of natural kind essentialism is compatible with the claim that kinds themselves have essential properties.

12 Reproductive isolation is the mechanism according to which “[…] the habits, habitat, physiology, and genetics of organisms that belong to a single species enable them to recombine their genes with those of others of the same species but not with those of any other species” (Matthen, 2009: 96).

13 Many other counterexamples can be found in Bird and Tobin (2017).

14 This does not preclude that concreteness can also be seen as an *extrinsic* property, especially when ‘being concrete’ is identified with ‘being located in spacetime’. However, this is not the kind of view we are interested in here, since, as has been argued above, becoming past must involve alterations in a thing’s *intrinsic* properties (to such an extent that it ceases to belong to its natural kind). Therefore, extrinsic concreteness cannot do the job.

15 Cf. Adams 1986, Keller 2004, Ingram 2016, 2018.

16 There are other views on which things are intrinsically different (when past) then they were when present. One can mention, for instance, Cameron (2015) Miller (2019).

17 Here, it is naively assumed that each present thing belongs to a *single* natural kind (because it seems to be the case in the classical Aristotelian view). But there seems to be no *a priori* reason to reject the idea that a thing may belong to several natural kinds. In any case, this would not affect the view that will be developed below.

18 Nonetheless, the question ‘What is it for something to become past?’ will find an informative answer in the next section: a thing (e.g., a person) that becomes past loses the properties that makes it belong to the kind to which it belonged when present (e.g., consciousness).

19 For a discussion of whether Plato is committed to bare particularism, see McPherran 1988.

20 The distinction between *thin* and *thick* particulars was popularized by David Armstrong (1997). However, according to Armstrong’s specific version of the substratum theory, particulars (which we encounter in ordinary experience) are states of affairs, and *thin* particulars exist only insofar as they can be “abstracted” from the *thick* particulars with which they are associated.

21 There are at least two ways to be a bundle theorist: “[*u*]*niversalist* bundle theorists take the properties or characteristics that are shared by objectively similar concrete particular objects to be universals. In contrast, *trope-theoretic* bundle theorists hold that that the properties or characteristics that are shared by objectively similar concrete particular objects are themselves particulars, viz., so-called *tropes*, *moments*, or *modes*. Tropes are construed by their proponents as particularized properties or individual qualities, e.g., the particular redness that inheres in a rose. Both types of bundle theorists view concrete particular objects as in some sense composed of, or constituted by, the properties that enter into a particular bundle; only they differ over whether the very same properties can be multiply located in distinct bundles at a single time” (Koslicki, 2018: § 1.4).

22 The bundle theorist is therefore committed to the controversial ‘Principle of Identity of Indiscernibles’ (PII), according to which necessarily, any two particulars that have all the same qualitative properties are the same particular.

23 As far as I understand it, this approximates the classical Davidsonian conception of events (cf. Davidson, 1970). For example, David Papineau has explicitly identified Davidson’s events with bare particulars: “[t]here are various possible ways of instituting the required relationship between mental and physical causes. If we think of events as bare particulars, we can say that each particular mental cause is the same event as the relevant physical cause (cf. Davidson’s ‘Mental Events’)” (1990: 66).

24 The expression comes from Cameron 2014.

25 Specifically, Smith admits degrees of existence: the more an event is temporally distant from the present, the less it is real. The result is that present particulars (or maximal existents) have nonrelational monadic properties and also stand in relations, while past (and future) particulars (i.e. what exists to less than the maximal degree) only stand in relations (cf. Smith, 2002: 132).

26 This is the Scotistic conception of thisness (after Duns Scotus who coined the term ‘haecceitas’, of which ‘thisness’ is intended to be a translation). This conception also stands in opposition to the ‘bundle’ conception of thisness, according to which a thing’s thisness is merely the conjunction of all of its qualitative properties (cf. Adams, 1979: 6–7).

27 An objection might be that only properties that are *parts* of a particular can characterize its nature. But, following Sider (2006: 390), I do not see *why* it should be so. After all, many properties a bare particular may instantiate are *intrinsic* properties. Moreover, even assuming that there could be *truly* bare particulars (i.e. particulars that instantiate no properties whatsoever), I do not see *what* could prevent us from saying that these particulars have a nature simply by *failing* to instantiate properties. In that sense, all *truly* bare particulars have the same nature, and that nature is “[…] exhausted by the fact that they instantiate no monadic universals” (Sider, 2006: 392).

28
*Truly* bare particulars might be useful in the metaphysics of spacetime, especially when considering *Ontic Structural Realism*, and in the metaphysics of mathematical entities (cf. Sider, 2006).

29 The distinction between *sparse* and *abundant* properties is due to David Lewis (1986: 59–69).

30 There are notable exceptions: Baxter 2001, Armstrong 2004.

31 The bundle theorist Laurie Paul overcomes this objection simply by denying that composition is unrestricted. According to her, there are no fusions containing the universals of *goldenness* and *mountainhood* as parts (cf. 2002: 579–580).

32 This objection can be overcome either by denying that the parthood relation is transitive, or by saying that particulars are ‘composed’ of universals in some sense that does not involve the usual notion of parthood.

33 The view that causal powers are necessarily connected with natural properties (which can be challenged, cf. *Humean contingentism*) is called ‘necessitarianism’ (cf. Tugby, 2020). The two most popular necessitarianist theories are ‘dispositional essentialism’ (cf. Bird, 2007) and ‘the identity theory’ (cf. Heil, 2003).

34 If it were the case, some might claim that we could not successfully refer to any powerless entity, such as *abstract entities* (e.g., Sherlock Holmes), which sounds suspicious.

35 As far as our intuitions are concerned, several reasons for adopting GBT (so conceived) rather than presentism can be found in Grandjean (2021b). In brief, the main reason is that only GBT provides a fundamental explanation as to why our *practices*, *emotions*, *attitudes*, and *observations* are time asymmetric.

## References

[CR1] Adams RM (1979). Primitive thisness and primitive identity. Journal of Philosophy.

[CR2] Adams, R, M. (1986). Time and Thisness. In *Midwest Studies in Philosophy *(Vol. 11, pp. 315–329)

[CR3] Aristotle (1999). *Physics*. R. Waterfield (trans.). Oxford University Press.

[CR4] Armstrong D (1989). A Combinatorial Theory of Possibility.

[CR5] Armstrong D (1997). A World of States of Affairs.

[CR6] Armstrong, D. (2004). How Do Particulars Stand to Universals?. In D. Zimmerman & K. Bennett (Eds.), *Oxford Studies in Metaphysics *(Vol. 1, pp. 139–154)

[CR7] Baker LR, Campbell J, O’Rourke M, Silverstein H (2010). Temporal reality. Time and Identity.

[CR8] Baxter D (2001). Instantiation as Partial Identity. Australasian Journal of Philosophy.

[CR9] Bailey, A. (2012). No bare particulars. In *Philosophical Studies*. (Vol. 158, pp. 31–41)

[CR10] Bergmann G (1967). Realism: A Critique of Brentano and Meinong.

[CR11] Bird A (2007). Nature’s Metaphysics: Laws and Properties.

[CR12] Bird, A., & Tobin, E. (2017). Natural Kinds. In E. Zalta (Ed.), *Stanford Encyclopedia of Philosophy. *https://plato.stanford.edu/entries/natural-kinds/. Accessed 21 Apr 2022.

[CR13] Black, M. (1952). The identity of indiscernibles. In *Mind *(Vol. 61, pp. 153–164)

[CR14] Bourne C (2002). When am I? A tense time for some tense theorists?. Australasian Journal of Philosophy.

[CR15] Bourne, C. (2006). *A Future for Presentism*. Oxford University Press.

[CR16] Braddon-Mitchell, D. (2004). How do we know that it is now now?. In *Analysis *(Vol. 64, pp. 199–203)

[CR17] Broad CD (1923). Scientific Thought.

[CR18] Burke, M. (1996). Tibbles the cat: A modern “Sophisma”. In *Philosophical Studies* (Vol. 84, pp. 63–74)

[CR19] Byrne C (2018). Aristotle’s Science of Matter and Motion.

[CR20] Cameron, R. (2014). Parts generate the whole, but they are not identical to it. In A. J. Cotnoir & D. L. M Baxter (eds.), *Composition as Identity *(pp. 90–108)

[CR21] Cameron R (2015). The Moving Spotlight: An Essay on Time & Ontology.

[CR22] Campbell K (1990). Abstract Particulars.

[CR23] Cohen S (1996). Aristotle on Nature and Incomplete Substance.

[CR24] Connolly, N. (2015). Yes: Bare particulars!. In *Philosophical Studies *(Vol. 172, pp. 1355–1370)

[CR25] Correia F, Rosenkranz SS (2018). Nothing to Come: A Defense of the Growing Block Theory of Time.

[CR26] Curtis, B., & Robson, J. (2016). *A Critical Introduction to the Metaphysics of Time*. Bloomsbury

[CR27] Dainton, B. (2010). *Time and Space*. 2nd edition. Routledge

[CR28] Davidson, D. ([1970] 2011). Mental Events. *Essays on Actions and Events* (pp. 207–224). Oxford University Press

[CR29] Evans G, Evans G (1985). Does tense logic rest on a mistake?. Collected Papers.

[CR30] Fine, K. (1994). Essence and modality. In *Philosophical Perspectives *(Vol. 8, pp. 1–16)

[CR31] Forrest, P. (2004). The real but dead past: A reply to Braddon-Mitchell. In *Analysis* (Vol. 64, pp. 358–362)

[CR32] Frege, G. ([1982] 1952). On sense and reference. In P. Geach & M. Black (Eds.), *Translations from the Philosophical Writings of Gottlob Frege*. Blackwell

[CR33] French, S. (2015). Identity and individuality in quantum physicis. In E. Zalta (ed.), *Stanford Encyclopedia of Philosophy. *https://plato.stanford.edu/entries/qt-idind/#PII. Accessed 21 Apr 2022.

[CR34] Gallois, A. (1990). Occasional identity. In *Philosophical Studies* (Vol. 58, pp. 203–224)

[CR35] Geach, P. (1973). The future. In *New Blackfriars* (Vol. 54, pp. 208–218)

[CR36] Geach, P. (1980). *Reference and Generality: An Examination of Some Medieval and Modern Theories*. 3rd edition. Cornell University Press

[CR37] Gibbard A (1975). Contingent Identity. Journal of Philosophical Logic.

[CR38] Grandjean, V. (2021a). How is the Asymmetry between the Open Future and the Fixed Past to be characterized? *Synthese, 198*, 1863–1886.

[CR39] Grandjean, V. (2021b). Symmetric and Asymmetric Theories of Time. *Synthese, 199*, 14403–14426.

[CR40] Grey, W. (1997). Time and becoming. In *Cogito* (Vol. 11, pp. 215–220)

[CR41] Grünbaum A (1967). Modern Science and Zeno’s Paradoxes.

[CR42] Heathwood, C. (2005). The real price of the dead past. In *Analysis* (Vol. 65, pp. 249–251)

[CR43] Heil J (2003). From an Ontological Point of View.

[CR44] Heller M (1990). The Ontology of Physical Objects.

[CR45] Husserl E (1964). The Phenomenology of Internal Time-Consciousness. Trans. J. S. Churchill.

[CR46] IASP (1994). Pain terms: a current list with definitions and notes on usage. H. Merskey & N. Bogduk (eds.), In *Classification of Chronic Pain* (2nd Edition, pp. 209–214). IASP Press

[CR47] Ingram, D. (2016). The virtues of thisness presentism. In *Philosophical Studies* (Vol. 173, pp. 2867–2888)

[CR48] Ingram D (2018). Thisness Presentism: An Essay on Time, Truth, and Ontology.

[CR49] Keller S, Zimmerman D (2004). Presentism and truthmaking. Oxford Studies in Metaphysics.

[CR50] KoslickiI, K. (2018). *Form, Matter, Substance*. Oxford University Press

[CR51] Kripke, S. (1980). *Naming and Necessity*. Blackwell

[CR52] Lewis D (1973). Counterfactuals.

[CR53] Lewis D (1983). New Work for a Theory of Universals. Australasian Journal of Philosophy.

[CR54] Lewis D (1986). On the Plurality of Worlds.

[CR55] Locke, J. ([1689] 1975). In P. Nidditch (Ed.), *An Essay concerning Human Understanding*. Clarendon Press

[CR56] Marcus, R. B. (1961). Modalities ad intensional languages. In *Synthese* (Vol. 13, pp. 303–322)

[CR57] Matthen, M. (2009). Chicken, eggs, and speciation’. In *Noûs* (Vol. 43, pp. 94–115)

[CR58] Mayr E (1942). Systematics and the Origin of Species.

[CR59] McPherran M (1988). Plato’s Particulars. Southern Journal of Philosophy.

[CR60] McTaggart, J. (1908). The unreality of time. In *Mind* (Vol. 17, pp. 457–474)

[CR61] Merricks J, Zimmerman D (2006). Goodbye growing block. Oxford Studies in Metaphysics Vol. 2.

[CR62] Meyer U, Dolev Y, Roubach M (2016). Consciousness and the present. Cosmological and Psychological Time.

[CR63] Miller K (2019). The cresting wave: A new moving spotlight theory. Canadian Journal of Philosophy.

[CR64] Moreland JP (2001). Universals.

[CR65] Myro G, Grandy R, Warner R (1985). Identity and time. Philosophical Grounds of Rationality: Intentions, Categories, Ends.

[CR66] Papineau, D. (1990). Why supervenience? In *Analysis* (Vol. 50, pp. 66–71)

[CR67] Paul, L. A. (2002). Logical parts. In *Noûs* (Vol. 36, pp. 578–596)

[CR68] Petkov, V. (2006). Is there an alternative to the block universe view?. In D. Dieks (Ed.), *The Ontology of Spacetime* (1st ed., pp. 207–228). Elsevier

[CR69] Plato. (2000). *Timaeus *(D. J. Zeyl, Trans). Hackett Publishing Co

[CR70] Putnam H (1967). Time and physical geometry. The Journal of Philosophy.

[CR71] Reimer, M. (2019). Reference. In E. Zalta (ed.), *Stanford Encyclopedia of Philosophy. *https://plato.stanford.edu/entries/reference/. Accessed 21 Apr 2022.

[CR72] Rettler, B., & Bailey, A. (2017). Object. In E. Zalta (ed.), *Stanford Encyclopedia of Philosophy. *https://plato.stanford.edu/entries/object/. Accessed 21 Apr 2022.

[CR73] Rietdijk. (1966). A proof of determinism derived from the special theory of relativity. In *Philosophy of Science *(Vol. 33, pp. 341–344)

[CR74] Sider T (2001). Four-Dimensionalism.

[CR75] Sider, T. (2006). Bare Particulars. In *Philosophical Perspectives* (Vol. 20, pp. 387–397)

[CR76] Sider, T. (2011). *Writing the Book of the World*. Oxford University Press.

[CR77] Simons P, Loux M, Zimmerman D (2003). Events. The Oxford Handbook of Metaphysics.

[CR78] Smith Q, Callender C (2002). Time and degrees of existence: A theory of ‘Degree Presentism’. Time, Reality and Experience.

[CR79] Tugby, M. (2020). Grounding theories of powers. In *Synthese*. 10.1007/s11229-020-02781-2

[CR80] Van Inwagen, P. (1981). The doctrine of arbitrary undetached parts. In *Pacific Philosophical Quarterly* (Vol. 62, pp. 123–137)

[CR81] Wiggins, D. (1968). On being in the same place at the same time. In *The Philosophical Review* (Vol. 77, pp. 90–95)

[CR82] Wildman, N. (2015). Load bare-ing particulars. In *Philosophical Studies* (Vol. 172, pp. 1419–1434)

[CR83] Williamson T (2013). Modal Logic as Metaphysics.

[CR84] Zimmerman D, Hawthorne J, Sider T, Zimmerman D (2008). The privileged present: Defending an “A-theory” of time. Contemporary Debates in Metaphysics.

[CR85] Zimmerman D, Callender C (2011). Presentism and the space-time manifold. The Oxford Handbook of Philosophy of Time.

